# Work hard, play hard: how sexually differentiated microglia work to shape social play and reproductive behavior

**DOI:** 10.3389/fnbeh.2022.989011

**Published:** 2022-09-12

**Authors:** Olivia Sullivan, Annie Vogel Ciernia

**Affiliations:** ^1^Djavad Mowafaghian Centre for Brain Health, University of British Columbia, Vancouver, BC, Canada; ^2^Graduate Program in Neuroscience, University of British Columbia, Vancouver, BC, Canada; ^3^Department of Biochemistry and Molecular Biology, University of British Columbia, Vancouver, BC, Canada

**Keywords:** microglia, sex, neurodevelopment, social behavior, sex hormones

## Abstract

Microglia are brain-resident immune cells that play a critical role in synaptic pruning and circuit fine-tuning during development. In the adult brain, microglia actively survey their local environment and mobilize inflammatory responses to signs of damage or infection. Sex differences in microglial gene expression and function across the lifespan have been identified, which play a key role in shaping brain function and behavior. The levels of sex hormones such as androgens, estrogens, and progesterone vary in an age-dependent and sex-dependent manner. Microglia respond both directly and indirectly to changes in hormone levels, altering transcriptional gene expression, morphology, and function. Of particular interest is the microglial function in brain regions that are highly sexually differentiated in development such as the amygdala as well as the pre-optic and ventromedial hypothalamic regions. With a focus on hormone-sensitive developmental windows, this review compares male and female microglia in the embryonic, developing, and adult brain with a particular interest in the influence of sex hormones on microglial wiring of social, reproductive, and disordered behavior circuits in the brain.

## Introduction

Microglia are brain-resident immune cells that compose 10%–15% of total brain cells (Kettenmann et al., 2011). *In vivo* imaging studies show homeostatic microglia as highly dynamic cells that survey the local brain environment (Davalos et al., 2005) and rapidly mobilize in response to injury or disease (Gomez-Nicola and Perry, 2015). Under inflammatory conditions, microglia become ameboid with retracted processes and a larger cell body (Nimmerjahn et al., 2005), which facilitates phagocytosis of cellular debris and the release of inflammatory cytokines and chemokines (Streit, 2000).

In addition to their immune-responsive activity, microglia carry out important age-specific functions (Thion and Garel, 2017). Microglia have been implicated as critical regulators of neuronal cell number, synapse formation, axon targeting, and interneuron lamination (Thion and Garel, 2017). These diverse functions are supported by recent advances in transcriptomic analysis which have revealed different microglial states that vary across developmental age, sex, and region (Hammond et al., 2019) of the healthy brain. One of the most rapidly expanding areas in microglial research is that of sex differences. This previously overlooked biological variable is emerging as a critical factor mediating differential microglial functions between the sexes in both health and disease.

**Figure 1:**
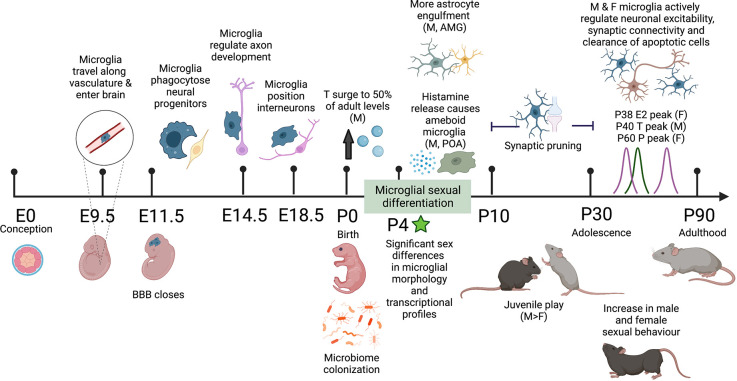
Schematic timeline of the sex differences observed in mouse microglial development and adult behavior. Microglia colonize the brain along the vasculature at E9.5 and fully colonize the brain by E11.5 upon closure of the blood-brain barrier (BBB). In embryonic development, microglia perform a range of organizational functions in the healthy brain. Male and female microglial transcriptomes are comparable throughout embyrogenesis but significantly diverge after birth, correlating with male testosterone (T) surge and microbiome colonization in both males and females. By P4 significant sex differences in microglial morphology are observed including more ameboid microglia and more microglial-astrocyte engulfment in males compared to females. Between P10 and P30 both male and female microglia undergo synaptic pruning. During this adolescent period, males display more juvenile play behavior. In early adulthood, there are observed increases in male and female sexual behavior along with peak levels of sex hormones: T in males and estradiol (E) and progesterone (P) in females. In the healthy adult mouse brain, microglia self-proliferate for the rest of life, continuing to sense and respond to their local environment and regulate neuronal excitability, synaptic connectivity, and clearance of apoptotic cells. Differential programming of microglia early in life alters their responses to immune challenges throughout life and may play a role in sex-biased prevalence rates in neurodevelopmental and neuropsychiatric disease. Created with https://biorender.com/.

Drawing on human and rodent studies, this review explores how sex influences the diversity of microglial roles throughout the lifetime with an emphasis on the role of sex hormones and brain environment in differentially influencing microglia function. During critical developmental windows, when the brain is highly pliable, microglia work to sculpt neuronal connectivity, often in a sex-specific manner. The work of microglia in regions of the brain that are highly differentiated between males and females directly influences sex-specific play behavior, social interactions, and reproductive behaviors (Figure 1). Disruption of microglial development can impair circuits and behaviors in a sex-specific manner, which may play a role in sex-biased prevalence of neurodevelopmental and neuropsychiatric disorders.

## Microglial Origins

During embryonic development, microglia are uniquely derived from erythromyeloid progenitors (Ginhoux et al., 2010). In mice, progenitors develop in the yolk sac and migrate through the vasculature to colonize the developing brain by E9.5, before the formation of the blood-brain-barrier (Alliot et al., 1999; Ginhoux et al., 2010; Kierdorf et al., 2013). In the absence of a functional circulatory system in NXC1 knockout mice, microglia fail to populate the brain (Ginhoux et al., 2010), highlighting the importance of the vasculature in embryonic microglial migration. Embryonic microglia also regulate axon development and interneuron positioning in the developing cortical layers (Squarzoni et al., 2014), however, to date the majority of these studies have not examined sex as a variable.

In humans, ameboid microglia are seen entering the human forebrain through the meninges, choroid plexus, and the ventricular zone between 4.5 and 5.5 gestational weeks (Verney et al., 2010). Once the progenitors enter the developing brain, they respond to a unique set of CNS-derived factors (Navascués et al., 2000; Gosselin et al., 2014, 2017) that drive a period of intense proliferation (Swinnen et al., 2013) and differentiation. Similar profiles are observed in rodents and non-human primates, suggesting evolutionary conservation across mammals (Dalmau et al., 2003; Billiards et al., 2006; Monier et al., 2007; Cunningham et al., 2013). Once the blood-brain barrier closes, microglia perpetually self-renew within the brain parenchyma, without replacement from peripheral sources, for the lifetime of the organism (Ajami et al., 2007).

## Microglia in The Developing Postnatal Brain

In early postnatal development, microglia are critical for neural circuit formation through synaptic pruning. In classic studies of the retinogeniculate circuit, Stevens and colleagues demonstrated that microglia phagocytose synaptic inputs from the retina during the first postnatal week. In this system, less-active synapses are tagged with complement proteins and subsequently targeted by microglia for engulfment (Schafer et al., 2012). Neural activity guided microglial pruning appears critical for proper circuit development in other brain regions as well, including the visual cortex (Tremblay et al., 2010), hippocampus (Paolicelli et al., 2011), striatum (Kopec et al., 2018), and frontal cortex (Mallya et al., 2019; Schalbetter et al., 2022). However, the open critical window in which pruning occurs appears to vary by brain region and is sensitive to individual experience. Microglial pruning can also be neuronal type specific, with a subset of GABA_B_ receptor expressing microglia selectively pruning GABAergic interneurons during cortical development (Favuzzi et al., 2021). Interestingly, microglia can also induce the formation of post-synaptic filopodia, potentially playing a role in synapse *formation* (Weinhard et al., 2018). Increasingly, studies are recognizing the importance of examining microglial pruning in the context of both sexes, often revealing sex-specific differences in critical windows and neuronal targets (Kopec et al., 2018; Rosin et al., 2021; Bolton et al., 2022).

## Microglia in The Adult Brain

In the healthy adult brain, microglia adopt a ramified morphology consisting of a small cell body and highly branched protrusions (Swinnen et al., 2013). In the absence of damage or infection, microglia actively survey their local brain environment. Each microglial cell appears to have a home territory that it monitors for damage or infection (Kettenmann et al., 2011). Microglia make regular transient contacts with neuronal synapses, astrocytes, and other brain cells (Nimmerjahn et al., 2005; Wake et al., 2009). These transient contacts likely contribute to healthy microglial regulation of neuronal excitability, synaptic activity, connectivity, neurogenesis, and clearance of apoptotic cells. For example, microglia can regulate hippocampal long-term potentiation (Costello et al., 2011; Rogers et al., 2011), a measure of synaptic plasticity and long-term memory. Microglia are also critical regulators of adult neurogenesis through targeted phagocytosis of apoptotic cells in the subgranular zone (Sierra et al., 2010). Microglia also clear naturally dying cells in the cerebellum (Ayata et al., 2018). Microglia can dampen neuronal activity by converting neuronal activity induced extracellular ATP to adenosine. The resulting increase in adenosine suppresses neuronal activity (Badimon et al., 2020) and failure in this system results in aberrant neuronal activity and seizures.

## Sex Hormones and Sexual Differentiation of The Brain

There is a growing appreciation for the role of the immune system, specifically microglia, in the sexual differentiation of the brain (McCarthy, 2020). Male and female microglia differ across development: there are notable sex differences in the number, function, and transcriptome of microglia in various brain regions and in disease (Hanamsagar et al., 2017; Guneykaya et al., 2018; Rahimian et al., 2019; Delage and Cornil, 2020; Han et al., 2021). These sex differences arise and contribute to the sexual differentiation of the brain beginning in embryogenesis.

The embryonic brain begins as bipotential, capable of adopting female or male typical phenotypes. In mammals, sex is largely determined by the presence of the Y chromosome gene Sry (Sex-determining region of the Y) that encodes for testis-determining factor protein (Tdf). In the presence of Sry, testes develop and secrete gonadal steroid hormones which masculinize the brain. In the absence of Sry, ovaries and associated female phenotypes develop (Lenz et al., 2012). In humans, sexual differentiation of the brain occurs between 8 and 24 gestational weeks and is further organized during periods of puberty when gonadal sex hormones (such as androgens, estrogens, and progesterone) surge (Bakker, 2019).

The bipotential of the early embryo is also reflected in the microglial transcriptome which is largely similar between males and females at E18 but significantly diverges during postnatal development (Hanamsagar et al., 2017; Thion et al., 2018). The transcriptional divergence between male and female microglia corresponds with the early postnatal hormone surge in males and with microbiome colonization upon birth (Thion et al., 2018) in both sexes. An absence of the gut microbiome during development more dramatically alters transcription and epigenetic regulation in male microglia compared to female. Altogether, this supports a model recently put forth by Bordt et al. (2020) and VanRyzin et al. (2020) in which both intrinsic regulation by sex chromosomes and extrinsic influences from sex hormones and the environment combine to shape sex-specific aspects of microglial development.

## Sex Hormones in Early Life

In male rodents, there is a surge in testicular androgen production beginning in the last few days of gestation that lasts until shortly after birth (Lenz and McCarthy, 2015). In primates, androgen surges occur from the end of the first trimester into the second trimester and again at birth (Lenz and McCarthy, 2015). During this window, aromatase catalyzes the conversion of testosterone into estradiol or dihydrotestosterone (Lephart, 1996). Estradiol (E2) acts as the primary driver of masculinization of the embryonic rodent brain (McCarthy et al., 2018). Interestingly, sex differences in microglia morphology and number manifest after the perinatal testicular androgen surge (Schwarz et al., 2012). These differences occur in multiple brain regions, including the hypothalamic medial preoptic area (POA; Lenz et al., 2013), amygdala (VanRyzin et al., 2019), and hippocampus (Nelson et al., 2017). By postnatal day (P) 4, sex has a significant effect on microglial morphology, specifically, within the parietal cortex, hippocampus, and amygdala where males show more ameboid microglia compared to females (Schwarz et al., 2012).

In addition to interacting with microglia directly, sex hormones can also influence the local brain microenvironment and indirectly impact microglia. For example, in male rats, aromatization of testosterone to estradiol in the POA causes nearby mast cells to release histamine, resulting in more ameboid microglia (Lenz et al., 2018). However, these sex differences naturally reverse by P30, with female rats showing more ameboid morphology than males in adulthood (Schwarz et al., 2012). In adult mice, females also show more ameboid microglial morphologies at baseline, resulting in LPS-induced shifts from highly branched to ameboid only in males (Hanamsagar et al., 2017).

## Sex Hormones During Adolescence and Adulthood

Microglia may also be critical for the active feminization of the brain and behavior. Although estradiol is responsible for masculinization of the brain early in life, prepubertal secretion of estradiol is required for sexual receptivity in females (Bakker et al., 2003). Microglia contain E2 receptors (Sierra et al., 2008) and thus are responsive to estradiol changes in the brain.

Microglia E2 receptors may differentially regulate inflammatory responses between the sexes. Estradiol has been shown to enhance microglial reactivity to immune challenges during puberty in the ventromedial nucleus (VMN) of the hypothalamus (Velez-Perez et al., 2020), a key brain region for female sexual behaviors (McCarthy, 2010).

Ovariectomized mice also show impaired induction of several immune genes in response to lipopolysaccharide (LPS) or live viral infection (Soucy et al., 2005). Conversely, in adult rats and mice systemic delivery of estrogen blocks LPS-induced changes in microglia morphology across several brain regions. The estrogen-mediated suppression of LPS response is absent in female ERα-null mice but intact in ERβ-nulls. ERα-null mice also developed aberrant microglial function with age in both males and females, suggesting important roles for ERα in microglial regulation in both sexes (Vegeto et al., 2003). Similar immune repressive effects of estrogens have been observed in rat microglial cultures (Vegeto et al., 2001). Together these studies support a complex role for ERα in both suppressing and augmenting microglial immune activity in the context of inflammation. Future work addressing the limitations of this work should employ genome wide transcriptomic approaches in combination with microglia specific and inducible models of ERα deletion to help clarify how estrogen modulates microglial activity across development and disease states.

Transcriptomic profiling of the adult whole brain microglia revealed differences in gene expression, with male microglia having a higher expression of genes related to inflammation and female microglia having a higher expression of genes associated with morphogenesis and cytoskeletal organization (Villa et al., 2018). Sex differences in gene expression were also found in hippocampal microglia (Hanamsagar et al., 2017; Villa et al., 2018). These sex differences in adulthood could be driven by sex hormone singling in the brain, sex chromosomes and/or sex specific programming during development. The expression of several target genes in female hippocampal microglia did not vary across the estrous cycle (Hanamsagar et al., 2017). Transcriptional profiles also appear resistant to sex-specific changes in the local brain environment by adulthood, as female mouse microglia transplanted into the male brain maintain a female-oriented gene expression profile (Villa et al., 2018). However, masculinization of female pups during *development* (postnatal week 1) resulted in microglial expression of several target genes that more closely resembled males than females (Villa et al., 2018), supporting the hypothesis that the effect of estrogens during early development may induce permanent sex-specific microglial transcriptional programs that are not sensitive to manipulations in the adult. Together, these studies support a critical developmental window in which both intrinsic and extrinsic factors can influence microglial development.

## Microglia and Behavior

It is widely accepted that early life experiences have lasting impacts on health and behavior later in adulthood. Early life changes in gonadal hormone levels have a long-lasting influence on sex-specific adolescent and adult rodent behaviors such as social behavior, aggression, alloparenting rough- and -tumble play, conspecific sniffing, and sexual behavior (Sisk and Zehr, 2005; Bale et al., 2010). Microglia respond to gonadal hormones through surface receptors and through sensing changes in the local brain environment. Interestingly, microglia have also been shown to mediate social (Kopec et al., 2018), cognitive (Cornell et al., 2022), and mood disorder (Branchi et al., 2014; Nelson and Lenz, 2017) behaviors in rodents. Since microglia are responsible for refining neural circuitry in the developing brain (Stevens et al., 2007), they may serve as a causal link in sex hormone-regulated behaviors.

## Social Behavior

A key study by VanRyzin et al. (2019) demonstrated that the P0 testosterone surge in male rats is responsible for changes in microglia that sculpt sexually differentiated social circuits in the rodent brain. They observed that male rats possessed more phagocytic microglia in the amygdala between P0 and P4 that were specifically engulfing newly proliferating astrocytes. Lower numbers of astrocytes in the medial amygdala correlated with increased juvenile play bouts in males compared to females, suggesting long-term impacts on the developing amygdala circuits mediating play behavior. In support of this hypothesis, treating females with testosterone at P0 led to increased phagocytic microglia in the amygdala and social play behaviors comparable to their male counterparts in adolescence. Furthermore, inhibiting complement dependent phagocytosis in the male amygdala between P0 and P2 increased the number of newborn cells at P4 and in adolescence decreased the levels of male social play to that of females (VanRyzin et al., 2019). Together this suggests a causal link between sex-specific microglial functions in amygdala development and sex-specific differences in play later in life.

Microglial synaptic pruning also shapes social behaviors. Transient depletion of frontal cortex microglia in adolescence resulted in disruptions in adult behaviors including an impaired ability to discriminate between a novel and familiar mouse (Schalbetter et al., 2022). These deficits were not specific to social behaviors as depleted mice also showed memory impairments. The behavioral deficits were linked to altered synaptic engulfment and subsequent reduced neuronal dendritic branching and synapse density as adults. The same depletion experiments conducted in adults did not produce deficits, defining a critical window for microglial pruning of synaptic circuits in the frontal cortex (Schalbetter et al., 2022). While this study was conducted only in males, Kopec et al. (2018) identified sex-specific microglial pruning windows underlying social behaviors. They demonstrated that microglial phagocytosis of dopamine receptors in the nucleus accumbens during key adolescent time windows shaped male rat social play behavior. In females, dopamine receptor elimination during adolescence was not mediated by microglial engulfment. However, blocking complement mediated phagocytosis did impact female social play, suggesting an alternative mechanism for microglial regulation of play in females. Together, these studies provide compelling evidence for sex-specific microglial mediated impacts on neural circuits underlying sex-differences in adolescent social behaviors. These findings may be highly relevant for neurodevelopmental disorders such as autism spectrum disorder (ASD) that are characterized by both microglial abnormalities and deficits in social interactions.

## Sexual Behavior

The net result of embryonic sexual differentiation is to set up the brain architecture for subsequent production of female or male typical social and reproductive behaviors after puberty (Arambula and McCarthy, 2020). The POA serves as an example region in which microglia play a direct role in controlling sexual differentiation and associated male sexual behavior. During early postnatal life, aromatization of testosterone into estradiol increases the production of prostaglandin E2 (PGE_2_) in the POA (Lenz et al., 2013). Microglia produce PGE_2_ and express PGE_2_ receptors (Minghetti et al., 1997), making them prime candidates for mediating POA masculinization. Studies treating female rats with estradiol or PGE_2_ before birth “masculinized” this area of the brain, increasing ameboid microglial numbers to those of males. Additionally, treating females with estradiol in early postnatal life, caused male-typical mating behaviors. Intriguingly, inhibition of microglia with co-treatment of minocycline prevented male copulatory behavior in the females, demonstrating a clear role for microglia in mediating the masculinization effects of PGE_2_ in the POA and the associated male sexual behaviors, in adulthood (Lenz et al., 2013).

## Brain and Behavior Disorders

One of the most consistent findings in post-mortem human brains across brain disorders is the altered expression of genes critical for neuronal and immune function (Voineagu et al., 2011; Gupta et al., 2014; Parikshak et al., 2016; Gandal et al., 2018; Vogel Ciernia et al., 2020). Transient pharmacological depletion of embryonic mouse microglia during gestation results in long-term, sex-specific impacts on behavior, including hyper-activity in juvenile females and anxiolytic behavior in adult females (Rosin et al., 2018). Similar depletion of rat microglia during the postnatal testosterone surge (P0–4) results in sex-specific alterations in nest seeking and ultrasonic vocalizations in pups, memory and anxiety-like behavior impairments in juveniles, and impairments in adult male sex-behaviors (VanRyzin et al., 2016). Together these studies and others like them, suggest that the removal of microglia during key developmental windows can have long-term consequences on the developing neuronal circuits and result in sex-specific impacts on behavior.

A growing body of literature suggests that a wide variety of environmental exposures are associated with an increased risk of neurodevelopmental disorders, including infection during pregnancy (Hallmayer et al., 2011; Kalkbrenner et al., 2014; Fang et al., 2015; Lee et al., 2015). Maternal immune activation (MIA) mouse models lead to long-lived changes in microglial phagocytosis activity (Fernández de Cossío et al., 2017), interneuron function (Thion et al., 2019), axon targeting (Squarzoni et al., 2014), transcription (Mattei et al., 2017; Vogel Ciernia et al., 2018), and morphology (O’Loughlin et al., 2017). For example, offspring from dams induced with allergic airway inflammation during pregnancy showed impaired social approach and increased repetitive behaviors, similar to both social and repetitive behaviors observed in ASD (Schwartzer et al., 2015). Microglia isolated from juvenile female offspring from this model showed altered transcription and epigenetic regulation indicative of enhanced microglial sensitivity to the brain environment (Vogel Ciernia et al., 2018).

Other maternal perturbations can also disrupt microglial development including exposures to drugs of abuse, air pollution, and maternal stress. For example, prenatal opioid exposure reduced adolescent microglial pruning of dopamine receptors in the nucleus accumbens specifically in male rats. This pruning deficit was associated with impaired opioid extinction in adult males but not females, suggesting impacts of early life drug exposure on microglial pruning may alter drug taking behaviors in adulthood (Smith et al., 2022).

Similar sex-specific vulnerabilities were identified in male mice exposed to diesel exhaust particles during gestation. Exposed males showed altered microglial morphology, increased microglia-neuron interactions, and increased inflammatory cytokine production. The male-specific alterations in microglial responses were consistent with male-specific behavioral and metabolic deficits (Bolton et al., 2012, 2013, Bolton et al., 2014), suggesting a sex-specific causal link between microglial dysfunction, altered brain development, and behavioral abnormalities in males.

Early life stress can also reprogram microglia in a sex-specific fashion. Offspring that experienced embryonic cold stress showed sex-specific alterations in the transcriptional profiles of embryonic hypothalamic microglia. Male microglia had more significantly upregulated genes than female microglia, rendering them more sensitive to maternal cold stress. Gestational cold stress impaired adult social preference in both sexes but was rescued by gestational microglial depletion only in males, suggesting that the adult social deficits are driven by non-microglial mechanisms in females (Rosin et al., 2021). Recent work from Block et al. (2022) combined prenatal air pollution exposure with postnatal maternal stress. They identified male-specific impairments in social behaviors that were linked to altered microglial pruning of developing thalamocortical synapses in the anterior cingulate cortex specifically in males. Inhibiting microglial phagocytosis within the anterior cingulate cortex during the critical developmental pruning window mimicked the pollution plus stress male phenotype, supporting the role of male-specific alterations in microglial function and behavioral impairments related to neurodevelopmental disorders (Block et al., 2022).

## Conclusions

The significance of understanding sex differences in the brain is becoming increasingly important with the growing prevalence of sex biased neurodevelopmental and neuropsychiatric disorders. Future therapeutic strategies should be guided by research into differential organization of the male and female brain, with a particular focus on microglia and sex hormones. With the recent advances in *in vivo* imaging and transcriptomics, the field is poised to understand how sex impacts microglial sculpting of brain circuits and behavior. The work highlighted here emphasizes the need to include both sexes in studies of microglia and provides the key rationale for sex-specific approaches to therapeutic development for neuroinflammation.

## Author Contributions

All authors listed have made equal direct intellectual contribution to the work and approved it for publication.

## Funding

This work was supported by the Canadian Institutes for Health Research (CRC-RS 950-232402 to AC and Canada Graduate Scholarship-Master’s to OS); Natural Sciences and Engineering Research Council of Canada (RGPIN-2019-04450, DGECR-2019-00069 to AC); Scottish Rite Charitable Foundation (21103 to AC); Brain and Behavior Research Foundation (Young Investigator Award 26784 to AC). The funders had no role in study design, data collection and analysis, decision to publish, or preparation of the manuscript.

## Conflict of Interest

The authors declare that the research was conducted in the absence of any commercial or financial relationships that could be construed as a potential conflict of interest.

## Publisher’s Note

All claims expressed in this article are solely those of the authors and do not necessarily represent those of their affiliated organizations, or those of the publisher, the editors and the reviewers. Any product that may be evaluated in this article, or claim that may be made by its manufacturer, is not guaranteed or endorsed by the publisher.
